# 639. Playing Keep Away—The Practice of Contact Precautions for Multidrug-Resistant Organisms (MDRO): A Survey of the Society for Healthcare Epidemiology of America (SHEA) Research Network and Affiliated US-based Hospitals

**DOI:** 10.1093/ofid/ofaf695.203

**Published:** 2026-01-11

**Authors:** K C Coffey, Trudy Grossman, David Banach, Anthony Harris, David C Hooper, Susan Huang, Erica S Shenoy

**Affiliations:** University of Maryland School of Medicine, Baltimore, MD; Combating Antibiotic-Resistant Bacteria Biopharmaceutical Accelerator (CARB-X), Boston, Massachusetts; UConn Health, Farmington, Connecticut; University of Maryland School of Medicine, Baltimore, MD; Massachusetts General Hospital, Boston, Massachusetts; University of California, Irvine School of Medicine, Irvine, California; Mass General Brigham, Boston, MA

## Abstract

**Background:**

Prevention of MDRO infection and transmission is a healthcare priority. Practices for screening, implementing and removing contact precautions (CP) are variable. The last SHEA Research Network (SRN) survey on this topic was almost a decade ago. Understanding current practices will inform future prevention strategies.

**Figure:**
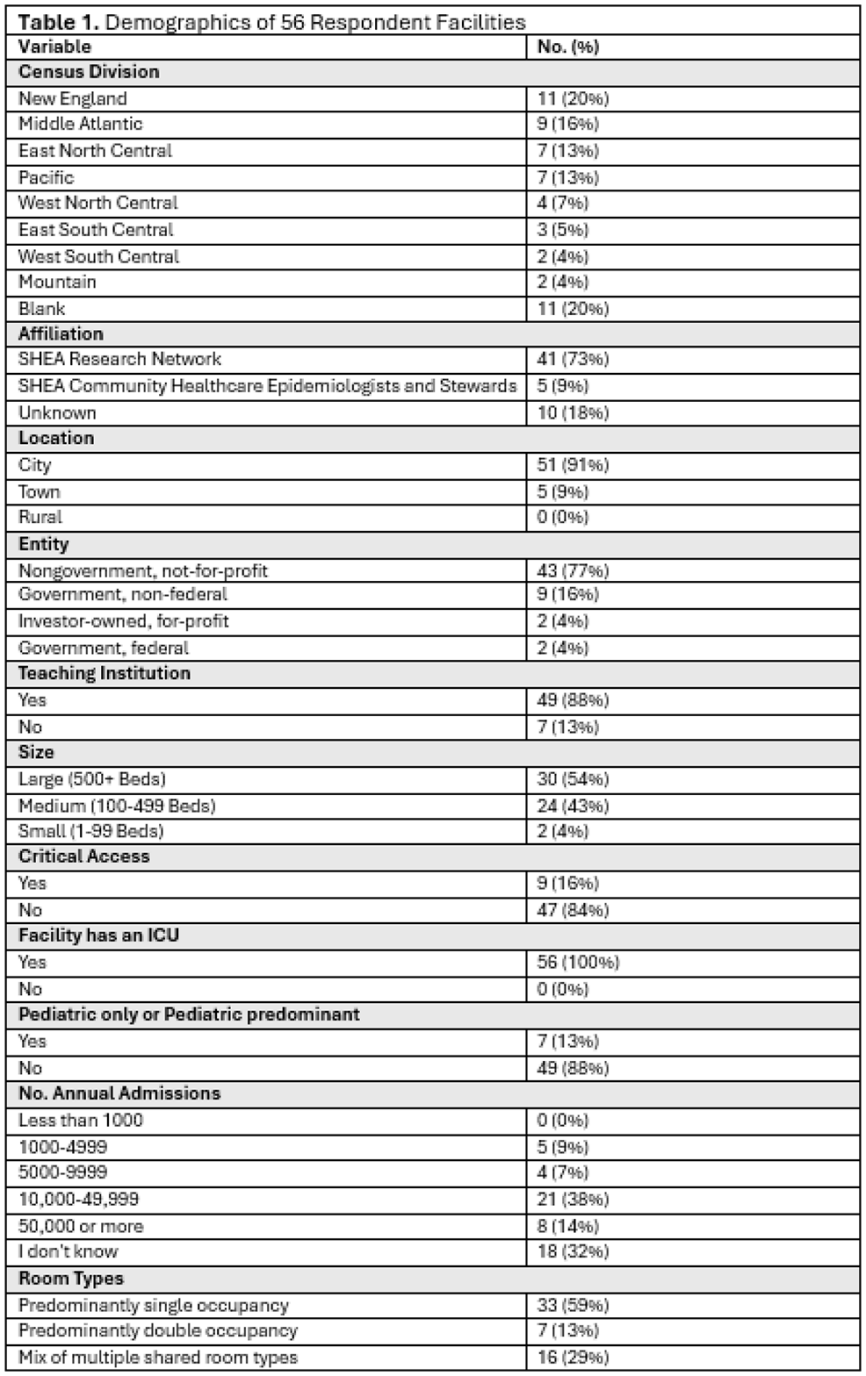

**Figure:**
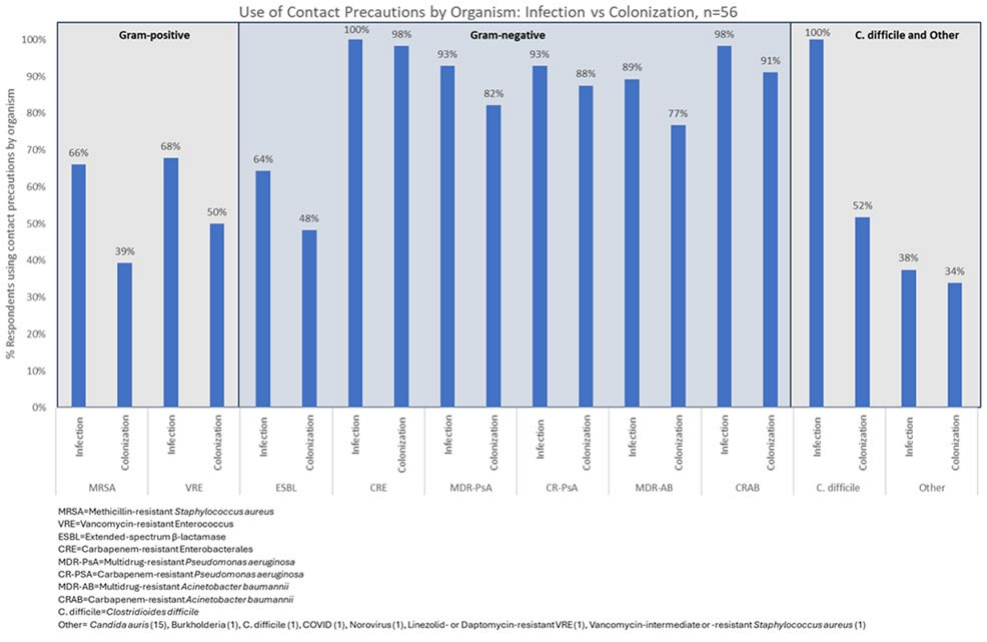

**Methods:**

We surveyed 134 SRN and SRN-affiliated US healthcare facilities on current MDRO practices. The survey was administered via REDCap from 1/7/25-2/25/25. Responses were de-identified and not linked to individuals or facilities. Survey responses were primarily categorical. Descriptive statistical analysis was performed in REDCap and Excel. This survey was considered nonhuman subjects research by the Mass General Brigham Institutional Review Board.

**Figure:**
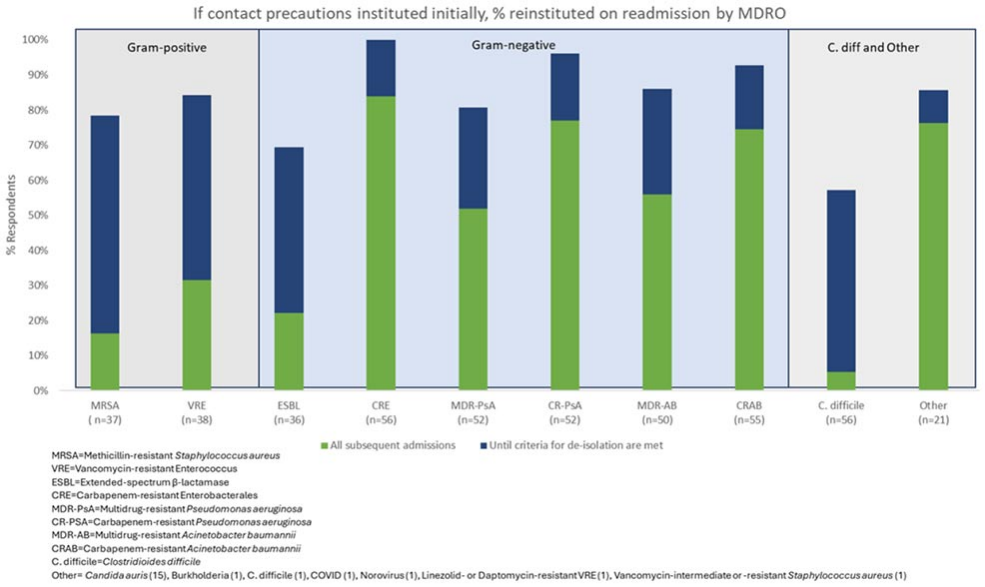

**Figure:**
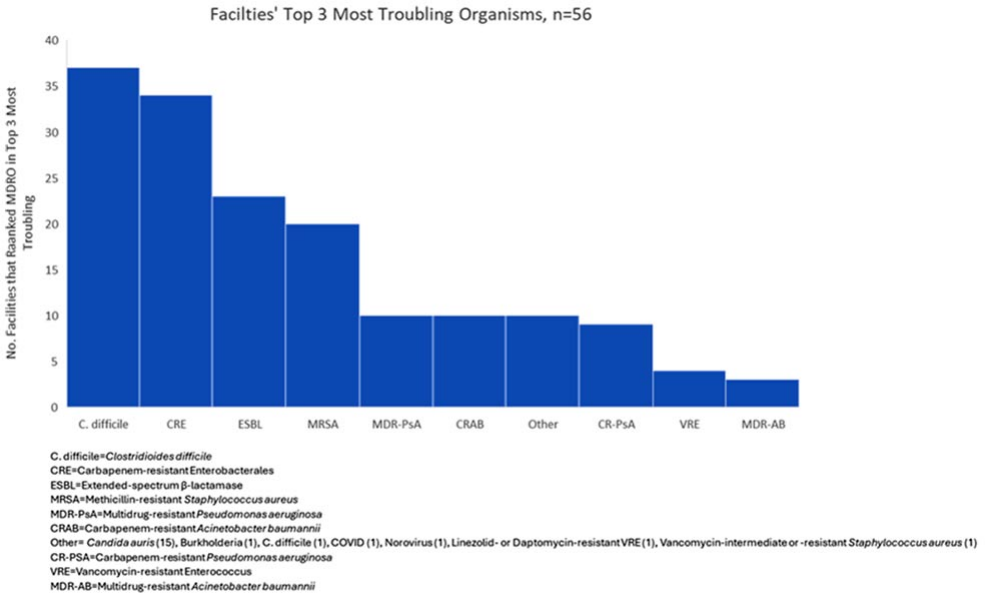

**Results:**

Of 134 facilities, 60 (45%) submitted the survey; 4 only answered demographic questions and were excluded from analysis. Demographics are shown in Table 1. All respondents reported using CP for > 1 MDRO, more for infection vs. colonization and gram-negative (GN) vs. gram-positive (GP) MDRO (Figure 1). On average, CP persist on subsequent admissions for GN (68%) compared to GP MDRO (24%) (Figure 2). Most facilities (70%) perform active surveillance for >1 MDRO, but only 16% employ preemptive CP. Respondents ranked methicillin-resistant *Staphylococcus aureus* (88%), *Clostridioides difficile* (86%) and extended-spectrum β-lactamases (ESBL) (84%) as common or very common. *C. difficile* (66%), carbapenem-resistant Enterobacterales (61%), and ESBL (41%) were the top 3 most troubling MDRO (Figure 3). Training/adherence (70%), private room availability (41%) and lack of evidence-based strategies to eradicate reservoirs (34%) were the top 3 identified barriers to infection prevention.

**Conclusion:**

Approaches to CP vary by infection vs. colonization, GP vs GN MDRO, and duration. This heterogeneity signals a need for better data on transmission risk by disease state, organism and time. With more data, professional organizations should guide facilities on tailoring MDRO prioritization, CP duration, and CP removal including time- or test-based protocols. Such guidance must address lack of private rooms and eradication of environmental reservoirs to overcome the major barriers identified in this survey.

**Disclosures:**

Anthony Harris, MD, MPH, UpToDate Wolters Kluwer Health: Infection control section editor

